# Endocarditis caused by *Aspergillus fumigatus* in a patient 9 months after COVID-19 infection recovery: a case report and review of the literature

**DOI:** 10.1186/s13256-023-04252-x

**Published:** 2023-12-19

**Authors:** Zeynab Yassin, Shokoufeh Hajsadeghi, Mohsen Taghavi Shavazi, Mahsa Fattahi, Koohyar Ahmadzadeh, Armita Farid, Yeganeh Karimi, Farnoosh Seirafianpour, Pegah Babaheidarian, Azadeh Goodarzi

**Affiliations:** 1 Antimicrobial Resistance Research Center, Institute of Immunology and Infectious Diseases, Iran University of Medical Sciences, Niayesh Street, Sattarkhan Avenue, Rasool Akram Medical Complex, Tehran, 1445613131 Iran; 2 Research Center for Prevention of Cardiovascular Disease, Institute of Endocrinology and Metabolism, Iran University of Medical Sciences, Niayesh Street, Sattarkhan Avenue, Rasool Akram Medical Complex, Tehran, 1445613131 Iran; 3 Department of Cardiology, Rasool Akram Medical Complex, Iran University of Medical Sciences, Niayesh Street, Sattarkhan Avenue, Rasool Akram Medical Complex, Tehran, Iran; 4 Center for Research and Training in Skin Diseases and Leprosy, Tehran University of Medical Sciences, Taleghani Ave, Nadery Ave, Tehran, Iran; 5 Physiology Research Center, Iran University of Medical Sciences, Tehran, Iran; 6 Razi Drug Research Center, School of Medicine, Iran University of Medical Sciences (IUMS), Iran University of Medical Sciences, Shahid Hemmat Highway, Tehran, 1449614535 Iran; 7 Rajaie Cardiovascular Medical and Research Center, Iran University of Medical Sciences, Tehran, Iran; 8 Pathology Department, Rasool Akram Medical Complex, Iran University of Medical Sciences, Tehran, Iran; 9 Department of Dermatology, Rasool Akram Medical Complex Clinical Research Development Center (RCRDC), School of Medicine, Iran University of Medical Sciences, Niayesh Street, Sattarkhan Avenue, Tehran, 1445613131 Iran; 10 Skin and Stem Cell Research Center, Tehran University of Medical Sciences, Tehran university of medical science, Tehran, 1445613131 Iran

**Keywords:** *Aspergillus*, Endocarditis, COVID-19, Fungal infection, Opportunistic infection, Review

## Abstract

**Background:**

*Aspergillus spp.* are among the fungal pathogens that can cause life-threatening infections in patients with a history of COVID-19.

**Case presentation:**

We present the case of a 58-year-old Iranian woman with post-COVID-19 *Aspergillus fumigatus* endocarditis complicated by numerous thromboembolisms. She underwent mitral valve replacement surgery and multiple lower extremity embolectomies and was treated with voriconazole, which led to her final recovery.

**Conclusions:**

*Aspergillus* endocarditis should be considered in any patient with suspected endocarditis who has a history of COVID-19 infection and does not respond to routine antibiotic and antifungal therapy, as COVID-19 interferes with proper immune function, and lack of underlying cardiac conditions and immunodeficiencies does not preclude the diagnosis. Culture and histopathological evaluation of vegetations and emboli, as well as PCR, can confirm the diagnosis. Early initiation of antifungal therapy and surgical removal of infected valves and emboli can improve prognosis in patients with *Aspergillus* endocarditis.

## Introduction

Since the start of the coronavirus disease-2019 (COVID-19) pandemic in December 2019, the severe acute respiratory syndrome coronavirus 2 (SARS-CoV-2) has rapidly spread worldwide, infecting over 410 million people and causing over 5 million deaths globally up to February 2022 [1]. There have been many reports of co-infections with different fungal and bacterial organisms in COVID-19 patients, which were associated with increased mortality [2, 3]. COVID-19 patients, particularly those in need of intensive care unit (ICU) admission, have several risk factors that can predispose them to invasive fungal infections, including intubation and mechanical ventilation, corticosteroid therapy, chronic respiratory diseases, and ongoing inflammation and cytokine storm [4]. Invasive candidiasis, mucormycosis, cryptococcosis, and aspergillosis have all been reported in COVID-19 patients, involving different organs such as the lungs, brain and heart [5].

Reports indicate that a significant number of individuals experience lingering symptoms following a COVID-19 infection, with percentages varying depending on the severity of the case. Hospitalized patients can exhibit ongoing symptoms in a range of 32.6% to 87%, while non-hospitalized patients report symptoms such as fatigue (37%) and cognitive impairment (30%) [6, 7]. In Wuhan, China, 76% of those previously infected still faced at least one symptom six months after discharge, and a study in Melbourne discovered persistent symptoms in 34% of individuals even after 45 weeks [8].

The prevailing theory points to an autoimmune process characterized by an overactive innate immune response and cytokine activation [9, 10]. Severe COVID-19 cases often display significantly elevated levels of pro-inflammatory cytokines, including interleukin 6, interleukin-1 beta, interleukin-2, interleukin-8, interleukin-17, granulocyte colony-stimulating factor, granulocyte–macrophage colony-stimulating factor, chemokine ligand 2, and chemokine ligand 10, as well as tumor necrosis factor alpha. This excessive cytokine activity is recognized as a “cytokine storm” [9].

The exact cause of this post-viral syndrome, which shares similarities with chronic fatigue syndrome (now termed post-viral fatigue syndrome or PVFS), remains unknown. Notably, it occurs in both hospitalized and non-hospitalized patients, seemingly independent of the initial infection's severity [11]. Some suggest that the underlying mechanism leading to neurological damage might be indirect, involving immune-related microvascular inflammation and thrombosis, which could explain the absence of viral markers. Importantly, the extent of proinflammatory markers appears to be linked to cognitive and behavioral changes [11].

*Aspergillus spp.* is among the fungal organisms that can complicate COVID-19 and lead to life-threatening infections in these patients. Some of the risk factors for aspergillosis in these patients include corticosteroid use, chronic obstructive pulmonary disease, prolonged neutropenia, immunodeficiencies, impaired mucociliary clearance, and other structural lung diseases [5, 12].

Most of the reported co-infections with *Aspergillus spp.* in COVID-19 patients are pulmonary infections. In a study from Germany, invasive COVID-19-associated pulmonary aspergillosis was found in 5 out of 19 patients who had moderate to severe acute respiratory distress syndrome (ARDS) [13]. In another study from Spain, 3.3% of ICU-admitted COVID-19 patients were diagnosed with COVID-19-associated pulmonary aspergillosis. These patients were mostly immunocompetent, had ARDS, and received corticosteroid therapy. Their mortality rate was 100% [14].

In this article, we present the first case of post-COVID-19 *Aspergillus fumigatus* endocarditis to our knowledge, complicated by numerous thromboembolisms.

## Case presentation

A 58-year-old Iranian woman was admitted to a secondary healthcare center complaining of paresthesia and weakness in her left lower limb which had begun abruptly on the night before admission. Her past medical history was notable for rheumatoid arthritis (with a drug history of 5 mg of prednisolone every other day and 200 mg of hydroxychloroquine taken daily) and a hospital admission due to COVID-19 nine months ago. The patient did not report any history of heart diseases, coagulation disorders and had not received any COVID-19 vaccinations. The patient did not report any history of heart diseases, or coagulation disorders and had not received any COVID-19 vaccinations. The patient underwent color Doppler ultrasonography of the lower limbs at the mentioned center and was diagnosed with arterial thrombosis of the left lower limb. She was referred to our hospital for further treatment.

On admission, the patient’s vital signs showed no fever, a blood pressure of 130/80 mmHg and a heart rate of 95 beats per minute. Physical examination revealed that the left lower limb was noticeably colder than the right lower limb, had much weaker distal pulses, and had an O2 saturation of 85% as opposed to 95% in the right lower limb.

Initial laboratory results showed a slightly elevated leukocyte count of 10.5 × 103/mm^3^ (normal 4–10 × 103/mm^3^), a hemoglobin level of 9.3 g/dl (normal 12–16 g/dl), thrombocytopenia with a count of 95 × 103/mm^3^ (normal 140–440 × 103/mm^3^), normal liver function tests, increased lactate dehydrogenase (LDH: 891 U/L, normal 225–500 U/L), increased C-reactive protein (CRP: > 24 mg/L, normal < 6 mg/L), increased erythrocyte sedimentation rate (ESR: 73 mm/hour, normal < 15 mm/hour), and an abnormal coagulation panel (INR: 1.16, normal 1–1.1; PT: 15.5, normal 12–13; PTT: 62.2, normal 25–45) (Table 1).

**Table 1:** Laboratory findings

Laboratory indicators	In this patient	Normal range
Leukocyte count	10.5 × 10^3^/mm^3^	4–10 × 10^3^/mm^3^
Hemoglobin level	9.3 g/dl	12–16 g/dl
Platelet count	95 × 10^3^/mm^3^	140–440 × 10^3^ /mm^3^
Lactate dehydrogenase	891 U/L	225–500 U/L
C-reactive protein	> 24 mg/L	< 6 mg/L
Erythrocyte sedimentation rate	73 mm/hour	< 15 mm/hour
INR	1.16	1–1.1
PT	15.5	12–13
PTT	62.2	25–45

*PTT* Partial Thromboplastin Time, *PT* Prothrombin Time, *INR* International Normalised Ratio, *mm/hr* millimeters per hour, *mg/L* milligrams per Liter, *mm* millimeter, *g/dl* grams per deciliter, *U/L* Units per Litre

The patient underwent a CT angiography of the lower limbs and was immediately sent to the operating room (OR) for aortic and iliofemoral thrombectomy. The patient was initially started on ceftriaxone and vancomycin (in the first 48 hour), which was changed to meropenem and vancomycin (and continued for 3 weeks). After the preliminary pathology results of the lower limb emboli revealed possible fungal hyphae, amphotericin B was also added to the treatment regimen (it was used for 5 days). The specimen was sent for further cultural evaluations.

The day after the surgery, the physical examination showed a significant improvement in circulation in the left lower limb, but the distal pulses of the right lower limb could not be palpated. Considering that acute signs of ischemia were not present in the right lower limb, possible thrombectomy of the right lower limb was postponed until the source of the emboli was found.

**Fig. 1:**
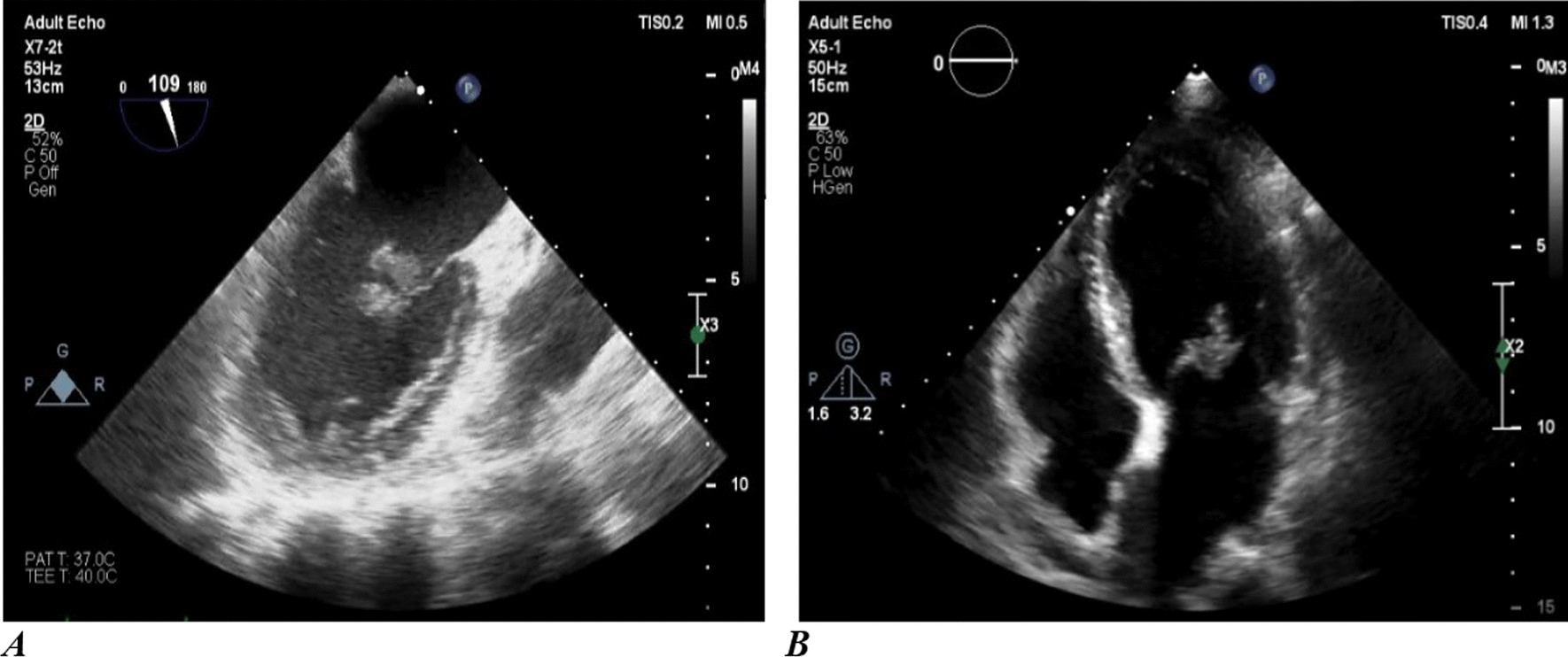
Echocardiogram of the patient showing a large mobile mass on tip of A2 scallop (with a length of roughly 16 mm) and another mass (6 mm × 7 mm) attached to mitral valve chorda in the left ventricle. Left picture **a** is a transesophageal echo in a 109-degree view, and the right picture **b** is a transthoracic echo in a four-chamber view

To find the source of the lower limb emboli, an echocardiogram was performed, which showed a large mobile mass on the tip of the A2 scallop [16 mm] and another mass (6 × 7 mm) attached to mitral valve chorda in the left ventricle (Fig. 1). The patient was scheduled for mitral valve replacement (MVR), which was performed one week after the left lower limb thrombectomy. The preliminary pathology report of the cardiac vegetation was also suggestive of a fungal infection, possibly aspergillosis or mucormycosis. Accordingly, amphotericin B therapy was continued. A coronary angiography was performed before the surgery, which yielded normal results.

A day after the MVR surgery, the patient’s right lower limb showed mottling and signs of ischemia. Therefore, a superficial right femoral artery thrombo-endarterectomy and right common femoral embolectomy were performed. After the surgery, the right lower limb was still colder, and its distal pulses were weaker than those of the opposite limb. Two days after the right lower limb thrombectomy, the patient began experiencing pain in the right leg and showed signs of mottling. Color Doppler ultrasonography of the right lower extremity arteries showed a cut-off external iliac artery. Angiography of the right lower limb confirmed the findings of the Doppler ultrasonography, and percutaneous old balloon angioplasty (POBA) was performed on the right external iliac artery. Due to the high thrombosis burden of the patient, no stent was implanted, and surgical thrombectomy was suggested in case of medical failure. The patient developed a hematoma at the site of angiography and was sent to the operating room (OR) for thrombo-endarterectomy of the right common femoral artery. The day after the surgery, both lower limbs were warm, and distal pulses were strong.

The patient did not develop any further complications during admission and underwent medical therapy with Amphotericin B while waiting for the final pathology reports. Eventually, culture results of the mitral valve came back, showing the growth of Aspergillus spp. PCR sequencing was performed to determine the subtype of the organisms, and the results revealed *Aspergillus fumigatus* (*A. fumigatus*) as the culprit pathogen. The organism showed resistance to Amphotericin B in the fungogram study conducted on the cultures. Consequently, the patient’s treatment regimen was changed to Voriconazole. On day 25 of treatment, Amphotericin B was switched to Voriconazole, and the total duration of antifungal therapy was 6 months. The patient was followed for 7 months after discharge, and no further complications developed.

## Discussion

Fungal endocarditis is an infrequent but lethal cause of endocarditis. After *Candida spp., Aspergillus spp.* is the second most common cause of fungal endocarditis [15, 16]. Immunosuppression, bone marrow transplantation, intravenous drug use, indwelling central venous catheters, parenteral nutrition, cardiac structural abnormalities, prosthetic heart valves, cardiovascular surgery, use of steroids and cytotoxic medications, and prolonged broad-spectrum antibiotic use are some of the predisposing risk factors for fungal endocarditis [16].

According to this study, a patient can develop fungal endocarditis even with very weak immunosuppression conditions. Despite expectations regarding fungal behavior, their sensitivity and resistance must be assessed with a fungogram to guide treatment.

As mentioned, if the fungogram had not been performed for this patient, she would not have been treated with amphotericin B, potentially causing more harm to the patient.

It seems that the chronic use of prednisolone significantly contributed to these conditions for this patient, but it is undoubtedly not without the influence of contracting COVID-19 and experiencing weakened immune system and coagulation disorders.

In one study, viral load in COVID-19 patients was positively correlated with lymphocyte suppression [17]. Another study found that in COVID-19 patients, especially critically ill ones, leukocyte subpopulations were markedly reduced, and adaptive and innate immune responses, as well as T and NK cell functions, were impaired [18].

Immune dysregulation induced by COVID-19 has been implicated in the pathogenesis of other invasive fungal co-infections such as mucormycosis [19]. One study reported a case of endocarditis with *Candida tropicalis* in a COVID-19 patient with a history of cardiac valve replacement [20]. Thus, COVID-19-associated ongoing immunosuppression could have led to *Aspergillus* endocarditis in our patient. Table 2 shows a summary of reported endocarditis cases with unusual pathogens in COVID-19 patients Additionally, SARS-CoV-2 and *A. fumigatus* were found to have common host–pathogen interaction targets. SARS-CoV-2 can recruit *Aspergillus* targets in addition to targets recruited by *A. fumigatus* itself. This can result in the development of invasive aspergillosis [21].

**Table 2:** Summary of reported endocarditis cases with unusual pathogens in COVID-19 patients

Authors	Patient age	Patient gender	Comorbidities	Interval between COVID-19 and endocarditis	Pathogen	Outcome
Arbune *et al.* [27]	23	Female	Rheumatic fever	Simultaneous	*Streptococcus gordonii*	Recovery
Davoodi *et al.* [20]	66	Male	Mitral and aortic valve replacement	Simultaneous	*Candida tropicalis*	Death
Mouzakitis *et al.* [41]	83	Female	Coronary artery disease, hypertension, diabetes mellitus	Simultaneous	*Staphylococcus lugdunensis*	Death
Finch *et al.* [42]	Not reported (young)	Male	Seborrheic dermatitis	Simultaneous	*Haemophilusparainfluenzae*	Recovery
Benmalek *et al.* [26]	76	Female	Diabetes mellitus	10 days	Coagulase-negative *Staphylococcus*	Recovery

Most of the reported COVID-19-associated *Aspergillus spp.* infections are pulmonary [22, 23]. During severe COVID-19, infected cells release danger-associated molecular patterns (DAMPs) which are endogenous danger signals that promote the immune response and inflammation leading to lung injury. Damage to the lung epithelium and inflammation both predispose to pulmonary aspergillosis [24]. There is also a report of a brain abscess caused by *A. fumigatus* in a COVID-19 patient who had received dexamethasone and multiple antibiotics [25]. Our patient is the first case of post-COVID-19 *Aspergillus* endocarditis to our knowledge. We performed a literature review of the reported COVID-19-associated extra-pulmonary *Aspergillus* infections, which is presented in Table 3. Although most *Aspergillus* infections occurred simultaneously or within a short time frame from the initial COVID-19 infection [2, 4, 5, 21, 22, 26, 27], there have also been reports of unusual infections occurring months after COVID-19 diagnosis, presumably due to COVID-19-induced chronic immune dysregulation as discussed earlier [6–11, 17]. Accordingly, *Aspergillus* infections can involve many organs including the lungs, brain, and heart in post-COVID-19 patients and should be considered during evaluations or infections in these patients, even long after the initial COVID-19 diagnosis.

**Table 3:** Summary of reported cases of COVID-19-associated extra-pulmonary Aspergillus infections

Authors	Patient age	Patient gender	Comorbidities	Interval between COVID-19 and *Aspergillus* infection	Infection type	Medical treatment	Outcome
De Villiers De La Noue *et al.* [25]	60	Male	None	35 days	Brain abscess	Voriconazole	Recovery
Makhdoomi *et al.* [43]	85	Male	None	7 months	Spinal abscess	Liposomal Amphotericin B	Recovery
Hosseinikargar *et al.* [44]	38	Male	Acute myeloid leukemia	Simultaneous	Rhinosinusitis	Fluconazole, Amphotericin B	Death
Hakamifard *et al.* [45]	35	Male	None	Simultaneous	Brain abscess	Voriconazole, Liposomal Amphotericin B	Death
Sahu *et al.* [46]	N/A	N/A	N/A	1–31 days	Endophtalmitis	Vitrectomy	Vision loss

N/A: not available

Previous studies have reported cases of *Aspergillus* endocarditis mostly in immunocompromised patients and patients with mechanical valves and other cardiac conditions [28–30], However, there have also been a few immunocompetent patients without underlying cardiac diseases who presented with this infection [31, 32]. One study reported a case of *Aspergillus* endocarditis and fulminant mediastinitis which led to death in an immunocompetent and otherwise healthy patient [33]. Our patient also had no history of underlying cardiac diseases or surgeries. Thus, it is important to consider possible fungal infections in mildly immunosuppressed patients with suspected endocarditis and without underlying cardiac conditions, especially in those with a history of COVID-19.

Based on existing literature, among patients diagnosed with infectious endocarditis, the dominant fungal species were *Aspergillus fumigatus*, accounting for 47.5% of cases, and Aspergillus flavus, which constituted 24.6% of the cases. Notably, a substantial 54% of the patients exhibited embolic events as part of their clinical presentation. Treatment strategies encompassed antifungal therapy in 90.2% of cases, cardiac surgery in 85.2% of cases, or a combination of both these approaches in 78.7% of cases. The mortality rate reached 52.5%. The immunosuppressed patients faced a significantly higher mortality rate in compared to their non-immunosuppressed counterparts, with rates of 59.4% versus 24.1%, respectively. Conversely, patients who underwent a combined treatment approach involving both cardiac surgery and azole therapy experienced a notably reduced mortality rate, standing at 28.1% compared to 65.5% for those who did not receive this dual therapy [34, 35]. In patients with *Aspergillus* endocarditis, vegetations are large and highly mobile, and peripheral emboli are common in the early stages [12]. Embolic phenomena are more common in *Aspergillus* endocarditis compared to bacterial endocarditis. The emboli can involve many organs, including brain, kidneys, spleen and lungs [36]. One study reported myocardial infarction caused by coronary artery embolism in a patient with *Aspergillus* endocarditis [36]. Lower extremity emboli similar to our patient's presentation have also been reported in previous studies [37, 38].

The diagnosis of *Aspergillus* endocarditis is obtained via 1,3-β-d-glucan testing, echocardiography, histopathologic evaluation and culture of the vegetations and emboli, as well as *Aspergillus* PCR. Transthoracic echocardiography (TTE) detects 90% of valvular vegetations [37]. The sensitivity of blood culture for the detection of *Aspergillus* infection is low. However, negative results do not exclude the diagnosis [39]. The most recent Infectious Diseases Society of America (IDSA) guidelines recommend using a combination of all the mentioned diagnostic tests for the detection of invasive aspergillosis [40]. In our patient, fungal vegetations were directly visualized on echocardiogram, and histopathological evaluation of both vegetations and emboli was suggestive of a fungal source of infection. The preliminary pathology results led to early initiation of antifungal therapy before the final confirmation of *A. fumigatus* infection with PCR. This timely treatment could be the cause of our patient’s good prognosis.

IDSA guidelines recommend early surgery in combination with antifungal treatment for Aspergillus endocarditis. A lipid formulation of amphotericin B or voriconazole is recommended as the initial treatment choice [40]. Our patient responded well to voriconazole in addition to MVR surgery and multiple embolectomies. However, her *A. fumigatus* strain showed resistance to amphotericin B, resulting in an initial lack of clinical response to treatment. Antifungal resistance has been reported among COVID-19-associated aspergillosis cases [41, 42]. This emphasizes the importance of early pathogen resistance profiling to optimize patients’ treatment regimen and prognosis.

## Conclusion

*Aspergillus* endocarditis should be considered in any patient, even with mild immunosuppression, who has a history of COVID-19 infection and does not respond to routine antibiotic and antifungal therapy, as COVID-19 interferes with proper immune function, and the lack of underlying cardiac conditions does not preclude the diagnosis. The mortality rate reported ranges from 50 to 68% in the literature. Culture and histopathological evaluation of vegetations and emboli and PCR can confirm the diagnosis. It is important to perform the fungogram test to check the sensitivity and resistance of fungi and choose the right treatment. Early initiation of antifungal therapy and surgical removal of infected valves and emboli can improve the prognosis in patients with Aspergillus endocarditis.

## Data Availability

Data resulted from this study are available from the corresponding author on reasonable request.

## Abbreviations

ICU: Intensive care unit
COVID-19: Coronavirus disease-2019
ARDS: Acute respiratory distress syndrome
SARS-CoV-2: Severe acute respiratory syndrome coronavirus 2
MVR: Mitral valve replacement
PCR: Polymerase chain reaction
TTE: Transthoracic echocardiography
IDSA: Infectious diseases society of America
